# Sequential Time to Positivity as a Prognostic Indicator in Persistent Candidemia

**DOI:** 10.1093/ofid/ofag619

**Published:** 2026-10-01

**Authors:** Marta Albanell-Fernández, Andrea Vergara, Marta Bodro, Mateu Espasa, Paula Juan, Josep Mensa, Montse Tuset, Sabina Herrera, Álex Soriano

**Affiliations:** Pharmacy Service, Division of Medicines, Hospital Clinic of Barcelona, University of Barcelona, Barcelona, Spain; Department of Physiological Science, School of Medicine, University of Barcelona, L'Hospitalet de Llobregat, Barcelona, Spain; Institut d’Investigacions Biomèdiques August Pi i Sunyer (IDIBAPS), Barcelona, Spain; Faculty of Medicine, University of Barcelona, Barcelona, Spain; Faculty of Medicine, University of Barcelona, Barcelona, Spain; Microbiology Department, Hospital Clinic of Barcelona, University of Barcelona, Barcelona, Spain; Institute of Global Health (ISGlobal), Barcelona, Spain; CIBERINFEC, CIBER in Infectious Diseases, ISCIII, Madrid, Spain; Institut d’Investigacions Biomèdiques August Pi i Sunyer (IDIBAPS), Barcelona, Spain; Faculty of Medicine, University of Barcelona, Barcelona, Spain; CIBERINFEC, CIBER in Infectious Diseases, ISCIII, Madrid, Spain; Infectious Diseases Department, Hospital Clinic of Barcelona, University of Barcelona, Barcelona, Spain; Faculty of Medicine, University of Barcelona, Barcelona, Spain; Microbiology Department, Hospital Clinic of Barcelona, University of Barcelona, Barcelona, Spain; Institute of Global Health (ISGlobal), Barcelona, Spain; Microbiology Department, Hospital Clinic of Barcelona, University of Barcelona, Barcelona, Spain; Institut d’Investigacions Biomèdiques August Pi i Sunyer (IDIBAPS), Barcelona, Spain; Infectious Diseases Department, Hospital Clinic of Barcelona, University of Barcelona, Barcelona, Spain; Pharmacy Service, Division of Medicines, Hospital Clinic of Barcelona, University of Barcelona, Barcelona, Spain; Institut d’Investigacions Biomèdiques August Pi i Sunyer (IDIBAPS), Barcelona, Spain; Faculty of Medicine, University of Barcelona, Barcelona, Spain; CIBERINFEC, CIBER in Infectious Diseases, ISCIII, Madrid, Spain; Institut d’Investigacions Biomèdiques August Pi i Sunyer (IDIBAPS), Barcelona, Spain; Faculty of Medicine, University of Barcelona, Barcelona, Spain; CIBERINFEC, CIBER in Infectious Diseases, ISCIII, Madrid, Spain; Infectious Diseases Department, Hospital Clinic of Barcelona, University of Barcelona, Barcelona, Spain

**Keywords:** blood cultures, candidemia, persistence, time to positivity

## Abstract

**Background:**

Persistent candidemia is associated with increased mortality, yet prognostic markers beyond initial blood culture time to positivity (TTP) remain poorly defined. While the TTP ratio between follow-up blood cultures (FUBCs) has shown prognostic utility in persistent *Staphylococcus aureus* bacteremia, its role in persistent candidemia has not been established. We aimed to evaluate the impact of changes in TTP across serial blood cultures in people with candidemia persisting for ≥48 hours and to assess their association with clinical outcomes.

**Methods:**

Single-center retrospective study including adult patients with candidemia and persistently positive FUBC ≥48 hours despite active antifungal therapy. Time to positivity and TTP ratio were analyzed according to clinical, microbiological, and outcome variables. The association with 30-day mortality was assessed using multivariable logistic regression.

**Results:**

Among 544 people with candidemia, 69 with persistent candidemia were included. The median TTP ratio was 1.4 (1.1–2.3). Patients with a shorter TTP ratio (<1.4) had numerically higher 30-day mortality, 7-day mortality, and in-hospital mortality, but in no cases was this statistically significant. The duration of antifungal exposure was not correlated with TTP prolongation. Thirty-day mortality was 36.2% and was associated in the multivariate model with septic shock, while catheter-related infection was protective.

**Conclusions:**

Overall mortality was numerically higher among those with shorter prolongation of FUBC TTP, although a statistical difference was not reached. No significant TTP prolongation was observed in sequential blood cultures despite active antifungal therapy. Further studies are needed to optimize therapeutic strategies against tolerant and persistent *Candida* strains.

**Main points:**

In people with persistent candidemia, a lower time-to-positivity (TTP) ratio (<1.4) had numerically higher mortality but was not statistically significant, likely due to the low number of events. Thirty-day mortality was associated in the multivariate model with septic shock, while catheter-related infection was protective. Duration of antifungal treatment was not associated with TTP prolongation. Further studies are needed to optimize management of persistent *Candida* strains.

## Graphical Abstract

**Graphical Abstract:**
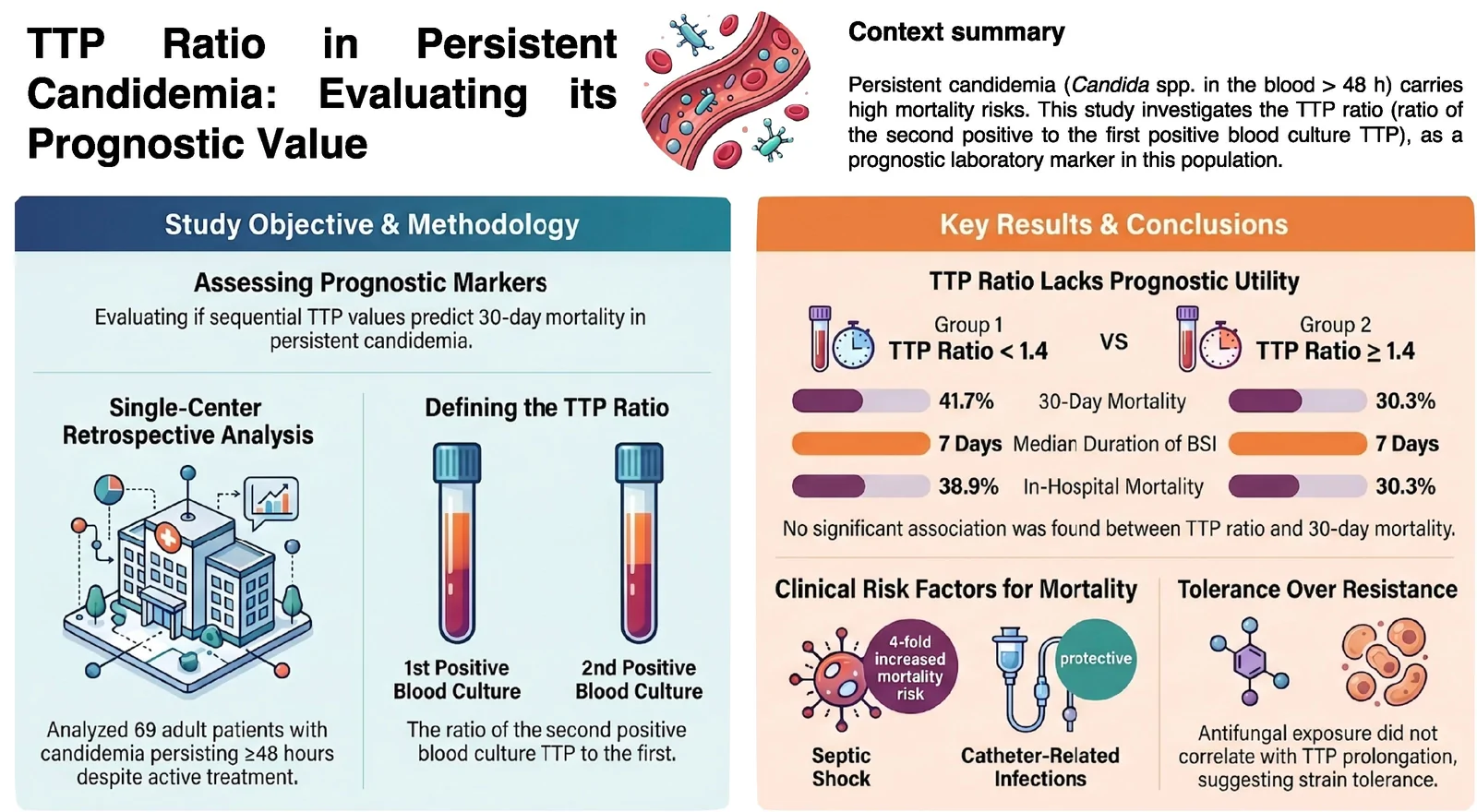

Persistent candidemia is relatively frequent, with reported rates ranging from 13.3% to 26.7% across studies [1–8]. Accumulating evidence from multicenter cohorts has demonstrated an association between positive follow-up blood cultures (FUBCs) and increased mortality [1, 2, 8]. Accordingly, the Infectious Diseases Society of America guidelines recommend obtaining FUBC daily or every other day until bloodstream clearance is documented [9]. In parallel, early removal of implicated intravascular catheters and prompt source control are key measures to reduce the risk of persistent infection [9, 10]. Time to positivity (TTP) of initial blood cultures (BCs) has been widely recognized as a surrogate marker of fungal burden and a relevant prognostic indicator in candidemia [11–13]. A short TTP (≤24 hours) has been independently associated with increased mortality [11] and longer TTP with higher survival rates [12], although significant interspecies variability exists. For instance, *Nakaseomyces glabratus* (*Candida glabrata*) typically shows longer TTP compared with *Candida albicans* and *Candida parapsilosis*, whereas *Candida tropicalis* is associated with shorter TTP values [13–16]. However, TTP is influenced by several preanalytical and clinical factors, including blood volume inoculated into the flasks, delays in sample processing, or the presence of antifungal therapy [17]; all of them should be considered when interpreting the TTP.

Beyond candidemia, TTP has been extensively investigated in *Staphylococcus aureus* bacteremia (SAB). In this setting, among patients with persistently positive FUBC, the TTP ratio (defined as the ratio of the second positive to the first positive BC TTP) has demonstrated prognostic significance. Specifically, a TTP ratio <1.5 has been independently associated with increased mortality and may help identify patients at elevated risk of complications, such as native valve endocarditis [18, 19]. Although candidemia and bacterial bloodstream infections differ substantially in their pathobiology, *Candida* spp. and *S. aureus* share relevant features, including biofilm formation and hematogenous dissemination. We hypothesized that, in people with persistent candidemia, prolongation of the TTP of FUBCs, reflecting a decrease in fungal burden, depends on an effective immune response and appropriate antifungal therapy. Consequently, we hypothesized that a higher TTP ratio would be associated with a better outcome.

Despite the established prognostic value of TTP in candidemia, the TTP ratio in people with persistent candidemia remains poorly defined. Therefore, the primary outcome was the change in TTP across serial BC in people with candidemia persisting for ≥48 hours and its association with clinical outcomes (30-day mortality, 7-day mortality, in-hospital mortality, duration of candidemia, duration of antifungal treatment, and length of hospital stay). Secondary outcomes included the influence of antifungal exposure on the TTP ratio and the factors influencing the 30-day mortality.

## METHODS

### Study Design and Setting

We conducted a retrospective study at an 800-bed tertiary university hospital in Barcelona (Spain). Data on all consecutive bloomsteam infections (BSIs) diagnosed between 2011 and 2025 were prospectively collected in a purpose-designed surveillance database. From this cohort, episodes of *Candida* spp. bloodstream infections were identified, and TTP, together with microbiological and relevant clinical variables (30-day mortality, duration of candidemia, antifungal treatment duration, and length of hospital stay), was analyzed.

Patients were eligible for inclusion if they had candidemia with positive FUBC occurring ≥48 hours after the initial culture and were receiving active antifungal treatment at the time of follow-up sampling. “Active antifungal treatment” was defined based on *in vitro* susceptibility of the isolated *Candida* spp. to the administered antifungal agent, in accordance with established breakpoints when available. Minimum inhibitory concentration (MIC) values were considered for this assessment. We excluded patients aged <18 years, those with polymicrobial candidemia, those without FUBC within 7 days of first positive BC, those who died before receiving active antifungal treatment, those with breakthrough candidemia under active antifungal treatment, and those with candidemia duration <48 hours. Follow-up blood culture in candidemia are routinely obtained 24–72 hours after the initial positive BC and continued every 3–4 days until culture clearance is achieved.

Collected variables included demographics, underlying conditions, risk factors for candidemia identified from the literature [10], septic shock at time of diagnosis, *Candida* species identification, source of candidemia, number of BC bottles collected, number of positive bottles for *Candida* spp., target antifungal therapy, and source control interventions.

### Microbiology

Volumes between 8 and 10 mL of blood samples were inoculated into aerobic and anaerobic bottles and processed using the BACTEC 9240 system from 2011 to 2013, and the BACTEC FX system (Becton–Dickinson, Block Scientific, Bellport, NY, United States) from 2013 onward. Incubation was continued for 5 days before bottles were reported as negative, and bottles were loaded into the instrument 24/7. The vials used were resin-containing BACTEC-Plus Aerobic/F and BACTEC-Plus Anaerobic/F or non–resin-containing BACTEC-Standard 10/Aerobic/F and BACTEC-Lytic/10/Anaerobic/F. When yeasts were observed on Gram stain, the positive BCs were subcultured onto Sabouraud and CHROMagar™ agar plates (BD BBL Stacker Plates, Heidelberg, Germany). Species identification was performed by Matrix-Assisted Laser Desorption/Ionization - Time of Flight (MALDI-TOF) mass spectrometry (Bruker).

### Definitions

*Candida* spp. BSI was defined as ≥1 BC positive for *Candida* spp. The candidemia source was determined based on clinical criteria and the isolation of the same strain from a clinical sample. Catheter-related candidemia was defined as growth of the same species both in peripheral BC and semi-quantitative tip culture. In our center, catheter-tip culture is a routine practice for people with candidemia whose catheters have been withdrawn. Intra-abdominal origin required clinical and radiological evidence of invasive candidiasis and/or positive cultures with *Candida* spp. obtained during surgery or by needle aspiration. Urinary tract origin was established if the same *Candida* spp. was isolated in the urine culture of a patient with a specific predisposing condition. Primary candidemia was defined as an infection with no apparent origin that did not match any other source criteria after adequate investigation.

Time to positivity was determined from the time interval between the start of incubation and the detection of microbial growth using an automated monitoring system (see microbiological procedures above). When multiple flasks were positive, only the shortest TTP was selected for the analysis. The TTP ratio was defined as the ratio of the TTP of the second positive BC to that of the first.

Microbiological clearance was defined as 3 consecutive days of negative BC, in accordance with recent global candidiasis guidelines [10].

High-grade candidemia was defined as the growth of *Candida* spp. in 2 or more sets of BCs or >50% of all collected flasks.

Septic shock was defined when the patient required vasopressors due to persistent hypotension despite fluid resuscitation at the time of candidemia diagnosis [20].

### Statistical Analysis

Categorical variables were presented as absolute numbers and percentages, and continuous variables were described as median and interquartile range (IQR). Associations between categorical variables were analyzed using Pearson's chi-square test or Fisher's exact test, as appropriate, while continuous variables with a nonnormal distribution were analyzed using the Mann–Whitney test. Patients were divided into groups according to the median TTP ratio.

The association between TTP ratio and 30-day mortality was assessed using logistic regression models, estimating odds ratios (ORs) with 95% confidence intervals (CIs). Time-to-positivity ratio was analyzed as a continuous variable and as a categorical variable, dichotomized according to its median value (P50). Because TTP differs across *Candida* spp., the median TTP was calculated separately for each species. A binary variable (TTP_50_) was then created to indicate whether the observed TTP was lower than the species-specific median, thereby accounting for interspecies variability in baseline TTP. We also explored additional cutoff points based on the species-specific distribution (P10, P25, P75, and P90) in sensitivity analyses.

All statistically significant covariates (*P* < .1) in univariate analysis were included in the multivariate model. Model performance and goodness-of-fit for the logistic regression models were evaluated using the area under the receiver operating characteristic curve (AUC) and the Hosmer–Lemeshow test, respectively.

Analyses were conducted with STATA/SE version 17.0 (StataCorp, College Station, TX, United States).

### Ethics Statement

The study was approved by the Ethics Committee of Hospital Clinic of Barcelona, Spain (HCB/2025/0796). The need for informed consent was waived due to the noninterventional, retrospective nature of the study.

## RESULTS

### Study Population

Five hundred forty-four patients had candidemia during the 14-year period, and 69 patients with positive FUBC met the study criteria and were included in the analysis (Figure 1).

**Figure 1.**
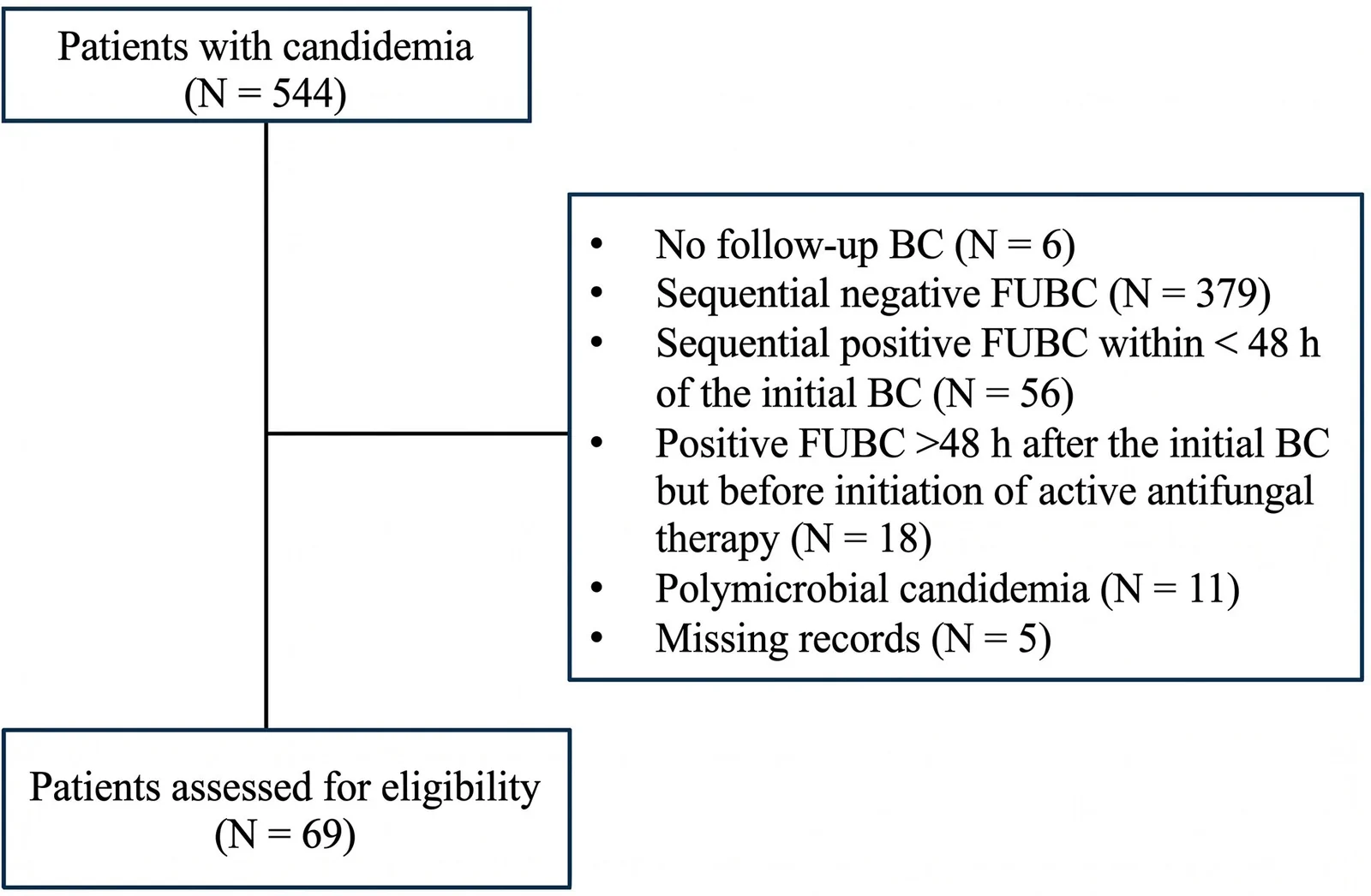
Study flow chart. Abbreviations: BC, blood culture; FUBC, follow-up blood culture.

Among patients, 38 (55.1%) were male, with a median (IQR) age of 65 (56–74) years. Thirty-five (50.7%) were admitted to the intensive care unit, and 23 (33.3%) had septic shock at diagnosis. The most frequent species was *C. parapsilosis* (n = 21, 30.4%), followed by *C. albicans* (n = 18, 26.1%). Catheter-related infection was identified in 31 patients (44.9%), and an abdominal source in 16 (23.2%). The overall median (IQR) TTP of the first BC was 27 hours (20.1–41), and FUBC was 45 hours (33–62). The second culture was obtained within a median (IQR) of 4 (3–6) days after the initial BC. The median (IQR) TTP ratio was 1.4 (1.1–2.3).

The main characteristics across study groups according to the sequential TTP/initial TTP ratio are summarized in Table 1. The only significant differences were observed in the TTP of the first positive BC, which was longer in the TTP ratio <1.4 group than in the TTP ratio ≥1.4 group (40.1 [25.4–48.0] vs 21.4 [17.0–30.3] h, respectively; *P* < .001). A catheter source was also more frequent among patients with a TTP ratio ≥1.4 (57.6% vs 33.3%; *P* = .043). Supplementary Table 1 summarizes the TTP according to source of candidemia and management.

**Table 1.** Comparison of the Main Characteristics Across Study Groups According to the Sequential TTP/Initial TTP Ratio

Characteristics	Second TTP/First TTP Ratio <1.4 (N = 36)	Second TTP/First TTP Ratio ≥1.4 (N = 33)	*P* Value
Sex (female), n (%)	17 (47.2)	14 (42.4)	.689
Age >65 y, n (%)	14 (38.9)	20 (60.6)	.071
Septic shock, n (%)	11 (30.6)	12 (36.4)	.609
Neutropenia, n (%)	4 (11.1)	3 (9.1)	.781
Previous surgery, n (%)	13 (36.1)	12 (36.4)	.983
Central venous catheter^a^, n (%)	27 (77.1)	27 (87.1)	.295
Total parenteral nutrition, n (%)	13 (36.1)	15 (45.5)	.430
Mechanical ventilation, n (%)	17 (47.2)	11 (33.3)	.241
Intensive care unit admission, n (%)	19 (52.8)	16 (48.5)	.722
Underlying conditions, n (%)			
Solid cancer	7 (19.4)	11 (33.3)	.189
Hematological malignancy	7 (19.4)	3 (9.1)	.222
Diabetes mellitus	6 (16.7)	12 (36.4)	.063
Chronic obstructive pulmonary disease	2 (5.6)	3 (9.1)	.572
Chronic kidney failure	6 (16.7)	9 (27.3)	.286
Liver disease	6 (16.7)	4 (9.1)	.592
Disorders requiring or causing immunosuppression	4 (11.1)	3 (9.1)	.781
Cardiovascular disease	13 (36.1)	16 (48.5)	.298
*Candida* species, n (%)			
*Candida albicans*	6 (16.7)	12 (36.4)	.063
*Nakaseomyces glabratus* (*Candida glabrata*)	7 (19.4)	4 (12.1)	.406
*Candida parapsilosis*	14 (38.9)	7 (21.2)	.111
*Candida tropicalis*	4 (11.1)	5 (15.2)	.619
*Pichia kudriavzevii* (*Candida krusei*)	3 (8.3)	2 (6.1)	.716
High-grade candidemia, n (%)	21 (58.3)	23 (69.7)	.327
Source of candidemia, n (%)			
Catheter related	12 (33.3)	19 (57.6)	.043
Abdominal	9 (25.0)	7 (21.2)	.710
Urinary	6 (16.7)	2 (6.1)	.169
Primary candidemia	6 (16.7)	5 (15.2)	.864
TTP of the first positive BC (h), median (IQR)	40.1 (25.4–48.0)	21.4 (17–30.3)	<.001
Initial treatment, n (%)			.353
Fluconazole	8 (22.2)	12 (36.4)	
Echinocandins	26 (72.2)	21 (63.6)	
Liposomal amphotericin B	2 (5.6)	0 (0)	
Time interval between the first positive BC and antifungal start (d), median (IQR)	1 (1–2)	1 (1–2)	.646
Adequate empirical treatment within 24 h of BC obtention, n (%)	16 (44.4)	14 (45.5)	.933
Time to source control (d), median (IQR)	2 (1–3)	2 (1–3)	.323
Source control within 24 h of BC collection, n (%)	11 (30.6)	14 (42.4)	.306
Change of antifungal during treatment, n (%)	20 (55.6)	19 (57.6)	.868

Abbreviations: BC, blood culture; TTP, time to positivity; IQR, interquartile range.

^a^Calculated for patients with a catheter (N = 66).

### Outcomes Stratified by Time-to-Positivity Ratio Groups

The mortality at 7 days (16.7% vs 9.1%, *P* = .351), at 30 days (41.7% vs 30.3%, *P* = .327), and in-hospital mortality (38.9% vs 30.3%, *P* = .454) were higher in the TTP ratio <1.4 group, although these differences were not statistically significant, probably due to the low number of patients evaluated. Supplementary Figure 1 shows the mortality rates (7- and 30-day mortality) stratified according to the TTP ratio. No statistically significant differences were observed in other outcomes (duration of *Candida*-BSI, antifungal treatment duration, or length of hospital stay) between groups stratified by TTP ratio (Table 2).

**Table 2.** Clinical Outcomes Stratified by Sequential-to-Initial Time-to-Positivity Ratio Groups

Parameter	Second TTP/First TTP Ratio <1.4 (N = 36)	Second TTP/First TTP Ratio ≥1.4 (N = 33)	*P* Value
In-hospital mortality	14 (38.9)	10 (30.3)	.454
7-d mortality^a^	6 (16.7)	3 (9.1)	.351
30-d mortality^a^	15 (41.7)	10 (30.3)	.327
Duration of BSI (d), median (IQR)^b^	7 (5–13.5)	7 (5–12)	.995
Total antifungal duration (d), median (IQR)	18 (14–30)	17.5 (12.5–24)	.360
Duration of hospital stay (d), median (IQR)	45 (30.5–85.5)	44 (23–76)	.313

Abbreviations: BSI, bloodstream infection; IQR, interquartile range; TTP, time to positivity.

^a^Mortality calculated from the first positive blood culture.

^b^A total of 63 patients achieved microbiological clearance, 32 in the TTP ratio <1.4 group and 31 in the TTP ratio ≥1.4 group.

### Influence of Antifungal Exposure on the Time-to-Positivity Ratio

To evaluate the impact of active antifungal exposure, Figure 2 illustrates the relationship between the duration of active antifungal therapy before the positive FUBC and the corresponding TTP ratio. Longer antifungal exposure was not associated with TTP prolongation or with higher TTP ratios.

**Figure 2.**
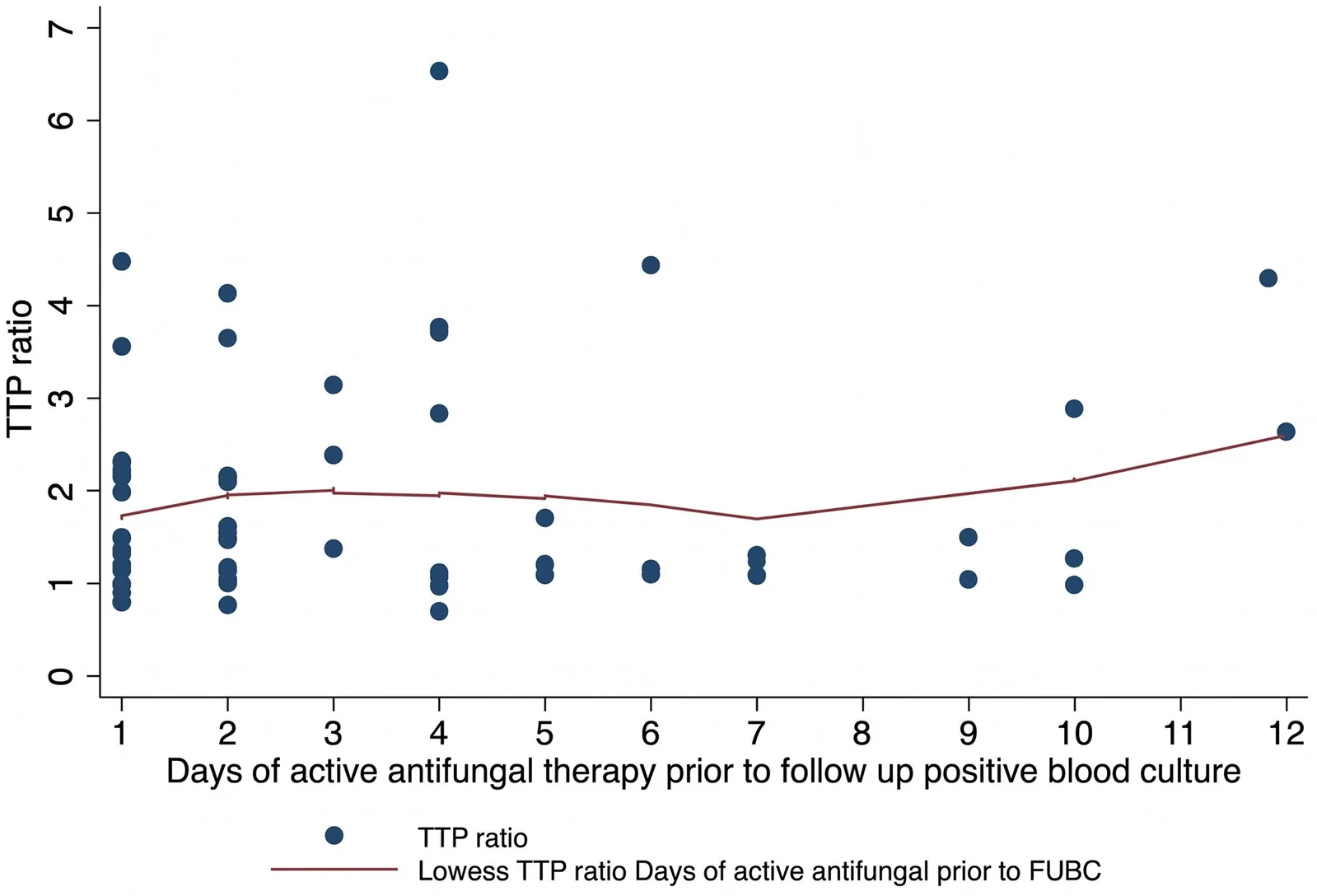
Graphical relationship between days of prior active antifungal therapy before the FUBC and the TTP ratio. Abbreviations: FUBC, follow-up blood culture; TTP, time to positivity.

### Risk Factors for Overall (30-Day) Mortality

The 30-day mortality was 36.2% (n = 25). Risk factors associated with all-cause mortality are shown in Table 3. The only risk factor for mortality was septic shock (OR 4.0, 95% CI 1.3–12.2, *P* = .014), while catheter-related candidemia was protective (OR 0.3, 95% CI .1–.9, *P* = .033) in the multivariable logistic regression. The model showed moderate discrimination, with an AUC of 0.7 (95%CI, .6–.8) and no evidence of poor model fit according to the Hosmer–Lemeshow test (χ^2^ = 1.34, *P* = .512). A TTP ratio <1.4 was not a significant risk factor for overall mortality in our study (OR 1.6, 95% CI .6–4.4, *P* = .328). As shown in Supplementary Table 2, modeling the TTP ratio as a continuous variable also showed no significant association with 30-day mortality (OR 0.98, 95% CI .94–1.03; *P* = .412). Similarly, no significant association with mortality was observed when alternative cutoff points (P10, P25, P75, and P90) were applied (Supplementary Table 3).

**Table 3.** Analysis of Parameters Associated With 30-Day Mortality Among People With Persistent Candidemia ≥48 h Receiving Active Antifungal Treatment

Parameter	Survived (N = 44)	Deceased (N = 25)	*P V*alue	Multivariate OR (95% CI)	*P* Value
Sex (female), n (%)	23 (52.3)	10 (40.0)	.536	…	…
Age >65 y, n (%)	19 (43.2)	15 (60.0)	.182	…	…
Septic shock, n (%)	10 (22.7)	13 (52.0)	.015	4.0 (1.3–12.2)	.014
Neutropenia, n (%)	4 (9.1)	3 (12.0)	.701	…	…
Previous surgery, n (%)	16 (36.4)	9 (36.0)	.976	…	…
Central venous catheter, n (%)	34 (82.9)	20 (80.0)	.765	…	…
Total parenteral nutrition, n (%)	17 (38.6)	11 (44.0)	.663	…	…
Mechanical ventilation, n (%)	15 (34.1)	13 (52.0)	.148	…	…
Intensive care unit admission, n (%)	19 (43.2)	16 (64.0)	.101	…	…
Underlying conditions, n (%)					
Solid cancer	12 (27.3)	6 (24.0)	.766	…	…
Hematological malignancy	7 (15.9)	3 (12.0)	.658	…	…
Diabetes mellitus	11 (25.0)	7 (28.0)	.785	…	…
Chronic obstructive pulmonary disease	2 (4.5)	3 (12.0)	.268	…	…
Chronic kidney failure	10 (22.7)	5 (20.0)	.792	…	…
Liver disease	6 (13.6)	4 (16.0)	.789	…	…
Disorders requiring or causing immunosuppression	7 (15.9)	3 (12.0)	.658	…	…
Cardiovascular disease	17 (38.6)	12 (48.0)	.450	…	…
*Candida* species, n (%)					
*Candida albicans*	12 (27.3)	6 (24.0)	.766	…	…
*Nakaseomyces glabratus* (*Candida glabrata*)	5 (11.4)	6 (24.0)	.161	…	…
*Candida parapsilosis*	16 (36.4)	5 (20.0)	.177	…	…
*Candida tropicalis*	5 (11.4)	4 (16.0)	.584	…	…
*Pichia kudriavzevii* (*Candida krusei*)	3 (6.8)	2 (8.0)	.856	…	…
High-grade candidemia, n (%)	27 (61.4)	17 (68.0)	.582	…	…
Source of candidemia, n (%)					
Catheter related	24 (54.6)	7 (28.0)	.036	.3 (.1–.9)	.033
Abdominal	10 (22.7)	6 (24.0)	.904	…	…
Urinary	4 (9.1)	4 (16.0)	.394	…	…
Primary candidemia	5 (11.4)	6 (24.0)	.177	…	…
TTP					
First TTP < TTP_50_	21 (47.7)	13 (52.0)	.733	…	…
TTP ratio < 1.4	21 (47.7)	15 (60.0)	.328	…	…
Intervention, n (%)					
Initial fluconazole treatment versus echinocandins	13 (29.6)	7 (28.0)	.892	…	…
Adequate empirical treatment within 24 h of BC collection	19 (43.2)	12 (48.0)	.699	…	…
Source control within 24 h of BC collection	18 (40.9)	7 (28.0)	.286	…	…

TTP_50_ is a binary variable based on the overall median TTP across all *Candida* species, categorizing patients according to whether their TTP was below or at/above the median.

Abbreviations: BC, blood culture; CI, confidence interval; OR, odds ratio; TTP, time to positivity.

Table 4 displays the differences in the TTP ratio according to the main *Candida* species.

**Table 4.** TTP Ratios Among the Main *Candida* Species

*Candida* Species	N Isolates	TTP Ratio (IQR)
*Candida albicans*	18	2.1 (1.3–2.3)
*Candida parapsilosis*	21	1.2 (1.0–1.5)
*Nakaseomyces glabratus* (*Candida glabrata*)	11	1.1 (0.8–3.6)
*Candida tropicalis*	9	1.5 (1.2–2.0)
*Pichia kudriavzevii* (*Candida krusei*)	5	1.1 (1.0–1.5)

Abbreviations: IQR, interquartile range; TTP, time to positivity.

## DISCUSSION

In this observational study of people with candidemia persisting for ≥48 hours despite active antifungal therapy, shorter prolongation of TTP in sequential BC, as a marker of poor microbiological response, was numerically associated with higher mortality, although it did not reach statistical significance. The TTP ratio was not significantly associated with 30-day all-cause mortality or other outcomes; however, these findings should be interpreted cautiously, as the limited sample size and number of mortality events may have resulted in insufficient statistical power to detect a clinically relevant association.

In SAB, the TTP in sequential FUBC appeared to correlate with improved clinical outcomes. Hsu et al [18] showed that, in people with SAB, a 1.5-fold increase in the second TTP was an independent predictor of mortality, consistent with Choi et al [21] who found that TTP in FUBC may predict complicated clinical courses in persistent SAB; notably, patients with decreasing TTP after source control and initiation of active antimicrobial therapy experienced higher 30-day mortality or developed secondary foci of infection. In candidemia, we have not been able to reproduce these findings, possibly due to the low number of patients included, although differences in the biological behavior of fungi after exposure to antifungals cannot be discarded.

Contrary to previous studies [11, 12], we found no association between shorter initial TTP and mortality in people with persistent candidemia. Differences in *Candida* species distribution and the use of common TTP thresholds across species in previous studies may partly explain these discrepant findings, as TTP and mortality vary substantially between species. In our cohort, the limited number of isolates per species precluded species-specific analyses; therefore, a species-specific association between initial TTP and mortality cannot be excluded.

Regarding the TTP ratio, in our cohort, it was numerically associated with higher mortality among patients with a TTP ratio <1.4 than among those with a ratio ≥1.4 (41.7% vs 30.3%, respectively), although this difference did not reach statistical significance (*P* = .327). Routine 5-day incubation of BC may have limited the detection of delayed growth in the FUBC; if a *Candida* isolate with an initial TTP of >48 to 72 hours subsequently showed a 2- or 3-fold increase in TTP, this delayed growth might not have been detected within the standard incubation period (5 days). This could have led to misclassification and underestimation of TTP prolongation. Longer incubation of FUBC could reduce this potential bias and might partly explain the absence of a statistically significant difference in mortality, as patients with greater TTP prolongation might be expected to have a more favorable prognosis.

The high mortality associated with persistent candidemia, even when the fungal load is reduced (longer TTP), can be explained in line with recent data showing that BC contain genetically diverse *C. albicans* and *N. glabratus* strains that differ in echinocandin tolerance and fitness [22–24]. Antifungal tolerance refers to the ability of a subpopulation of susceptible organisms to survive prolonged antifungal exposure without necessarily exhibiting an increased MIC or meeting established criteria for resistance [25]. Our findings (Figure 2) may be consistent with the persistence of tolerant subpopulations during antifungal treatment. In addition, it has been demonstrated that *C. albicans* isolates persisting in the bloodstream for ≥4 days despite antifungal treatment display enhanced adherence, filamentation, and biofilm formation in vitro, together with upregulation of key virulence-associated genes. Moreover, *C. albicans* isolates from persistent candidemia showed increased tolerance to innate immune responses, as macrophages kill nonpersistent isolates significantly more efficiently than persistent ones [26]. These findings may explain the high mortality observed in our cohort of people with persistent candidemia (36.2%), which is consistent with previous studies (32%–50.0%) [1, 2, 8], even after adequate source control, and despite evidence of partial reduction in fungal burden reflected by higher TTP ratios during antifungal therapy.

We observed marked interspecies differences in TTP ratios among *Candida* spp. The highest TTP ratio was observed in *C. albicans* (TTP ratio: 2.1), whereas *N. glabratus* showed the lowest ratio (TTP ratio: 1.1). These differences may reflect species-specific susceptibility to antifungal agents, potentially contributing to greater TTP prolongation in FUBP among *C. albicans* isolates, while *C. parapsilosis* (TTP ratio: 1.2) has been associated with reduced susceptibility to antifungals (echinocandins) compared with other species, particularly in the presence of biofilms. The lower TTP ratio observed in *N. glabratus* may be related to its greater genetic and phenotypic diversity [22], which could limit fungal burden reduction despite active antifungal therapy.

Several limitations should be acknowledged. First, this was a retrospective, single-center observational study and is therefore subject to inherent design-related biases. Second, the small sample size limits robust conclusions regarding mortality outcomes, particularly as the event occurred in only 25 patients (36.2%). Third, inclusion required survival long enough to undergo FUBC collection; this may have introduced survivor bias. Fourth, in our cohort, we did not assess the evolution or impact of antifungal therapy on TTP in the sequential positive FUBC; only the impact on the TTP of the first FUBC was analyzed. Fifth, the TTP of BC drawn at approximately the same time from a given patient may vary due to differences in sampled blood volume and delays between collection and incubation. This variability is likely greater in FUBC unless procedures are strictly standardized; nevertheless, a decrease in TTP remains uncommon in patients with adequate source control and appropriate antimicrobial therapy. Moreover, changes in BC instrumentation from BACTEC 9240 to BACTEC FX during the study period may have also influenced measured TTP values. However, this limitation reflects real-world clinical practice and underscores the challenges in interpreting TTP.

## CONCLUSIONS

In conclusion, the mortality of people with persistent candidemia is high, and numerically higher among those with shorter prolongation of FUBC TTP, although statistical significance was not reached given the limited sample size. Early candidemia detection remains important to facilitate prompt initiation of active antifungal therapy and potentially reduce the risk of persistent infection. Further multicenter studies are warranted to evaluate the prognostic utility of sequential TTP in persistent candidemia.

## Supplementary Material

ofag619_Supplementary_Data

## Supplementary Data

Supplementary materials are available at *Open Forum Infectious Diseases* online. Consisting of data provided by the authors to benefit the reader, the posted materials are not copyedited and are the sole responsibility of the authors, so questions or comments should be addressed to the corresponding author.

## References

[1] Tiseo G, Vena A, Bassetti M, et al Persistent candidemia caused by different *Candida* species: data from a multicenter contemporary cohort. J Infect 2025; 91:106586. 10.1016/j.jinf.2025.106586 40816720

[2] Sedik S, Egger M, Wolfgruber S, et al Risk factors for persistent candidemia and prognostic implications: results from the ECMM Candida III study. J Infect 2025; 91:106629. 10.1016/j.jinf.2025.106629 41106444

[3] Li Y, Gu C, Yang Y, et al Epidemiology, antifungal susceptibility, risk factors, and mortality of persistent candidemia in adult patients in China: a 6-year multicenter retrospective study. BMC Infect Dis 2023; 23:369. 10.1186/s12879-023-08241-9 PMC10233919 37264301

[4] Ala-Houhala M, Anttila V. Persistent vs non-persistent candidaemia in adult patients in 2007-2016: a retrospective cohort study. Mycoses 2020; 63:617–24. 10.1111/myc.13085 32274866

[5] Lin K-Y, Chen P-Y, Chuang Y-C, et al Effectiveness of echinocandins versus fluconazole for treatment of persistent candidemia: a time-dependent analysis. J Infect 2018; 77:242–8. 10.1016/j.jinf.2018.05.011 29932963

[6] Agnelli C, Valerio M, Bouza E, et al Persistent candidemia in adults: underlying causes and clinical significance in the antifungal stewardship era. Eur J Clin Microbiol Infect Dis 2019; 38:607–14. 10.1007/s10096-019-03477-3 30680572

[7] Kang SJ, Kim SE, Kim UJ, et al Clinical characteristics and risk factors for mortality in adult patients with persistent candidemia. J Infect 2017; 75:246–53. 10.1016/j.jinf.2017.05.019 28587831

[8] Albanell-Fernández M, Vergara A, Cardozo C, et al Defining the threshold duration of candidemia associated with poor outcomes: redefining persistent candidemia. J Infect 2026; 93:106798. 10.1016/j.jinf.2026.106798 42302914

[9] Pappas PG, Kauffman CA, Andes DR, et al Clinical practice guideline for the management of candidiasis: 2016 update by the Infectious Diseases Society of America. Clin Infect Dis 2016; 62:e1–50. 10.1093/cid/civ933 PMC4725385 26679628

[10] Cornely OA, Sprute R, Bassetti M, et al Global guideline for the diagnosis and management of candidiasis: an initiative of the ECMM in cooperation with ISHAM and ASM. Lancet Infect Dis 2025; 25:e280–93. 10.1016/S1473-3099(24)00749-7 39956121

[11] Kim S-H, Yoon YK, Kim MJ, Sohn JW. Clinical impact of time to positivity for *Candida* species on mortality in patients with candidaemia. J Antimicrob Chemother 2013; 68:2890–7. 10.1093/jac/dkt256 23825380

[12] Keighley C, Pope AL, Marriott D, et al Time-to-positivity in bloodstream infection for *Candida* species as a prognostic marker for mortality. Med Mycol 2023; 61:myad028. 10.1093/mmy/myad028 PMC10189360 37019819

[13] Albanell-Fernández M, Vergara A, Cardozo C, et al Time to positivity as a diagnostic tool for catheter-related candidemia: a species-specific analysis. Int J Infect Dis 2026; 170:108847. 10.1016/j.ijid.2026.108847 42229787

[14] Lai C-C, Wang C-Y, Liu W-L, Huang Y-T, Hsueh P-R. Time to positivity of blood cultures of different *Candida* species causing fungaemia. J Med Microbiol 2012; 61:701–4. 10.1099/jmm.0.038166-0 22245787

[15] Bordallo-Cardona MÁ, Sánchez-Carrillo C, Muñoz P, Bouza E, Escribano P, Guinea J. Growth kinetics in *Candida* spp.: differences between species and potential impact on antifungal susceptibility testing as described by the EUCAST. Med Mycol 2019; 57:601–8. 10.1093/mmy/myy097 30339238

[16] Fernandez J, Erstad BL, Petty W, Nix DE. Time to positive culture and identification for *Candida* bloodstream infections. Diagn Microbiol Infect Dis 2009; 64:402–7. 10.1016/j.diagmicrobio.2009.04.002 19446982

[17] Lamy B . Blood culture time-to-positivity: making use of the hidden information. Clin Microbiol Infect 2019; 25:268–71. 10.1016/j.cmi.2018.12.001 30580034

[18] Hsu M-S, Huang Y-T, Hsu H-S, Liao C-H. Sequential time to positivity of blood cultures can be a predictor of prognosis of patients with persistent *Staphylococcus aureus* bacteraemia. Clin Microbiol Infect 2014; 20:892–8. 10.1111/1469-0691.12608 24612429

[19] Comba IY, Go JR, Vaillant J, et al Sequential time to positivity as a prognostic indicator in *Staphylococcus aureus* bacteremia. Open Forum Infect Dis 2024; 11:ofae173. 10.1093/ofid/ofae173 PMC11010313 38617074

[20] Evans L, Rhodes A, Alhazzani W, et al Surviving sepsis campaign: international guidelines for management of sepsis and septic shock 2021. Intensive Care Med 2021; 47:1181–247. 10.1007/s00134-021-06506-y PMC8486643 34599691

[21] Choi SH, Chung JW. Time to positivity of follow-up blood cultures in patients with persistent *Staphylococcus aureus* bacteremia. Eur J Clin Microbiol Infect Dis 2012; 31:2963–7. 10.1007/s10096-012-1648-0 22644058

[22] Badrane H, Cheng S, Dupont CL, et al Genotypic diversity and unrecognized antifungal resistance among populations of *Candida glabrata* from positive blood cultures. Nat Commun 2023; 14:5918. 10.1038/s41467-023-41509-x PMC10516878 37739935

[23] Fleres G, Cheng S, Badrane H, et al Blood cultures contain populations of genetically diverse *Candida albicans* strains that may differ in echinocandin tolerance and fitness. J Infect Dis 2026; 233:560–72. 10.1093/infdis/jiaf495 PMC13017712 41026105

[24] Rosenberg A, Ene IV, Bibi M, et al Antifungal tolerance is a subpopulation effect distinct from resistance and is associated with persistent candidemia. Nat Commun 2018; 9:2470. 10.1038/s41467-018-04926-x PMC6018213 29941885

[25] Yang F, Berman J. Beyond resistance: antifungal heteroresistance and antifungal tolerance in fungal pathogens. Curr Opin Microbiol 2024; 78:102439. 10.1016/j.mib.2024.102439 PMC7616270 38401284

[26] Ribeiro FC, Kemmerich KK, Gonçale JC, et al *Candida albicans* recovered from persistent candidemia exhibits enhanced virulence traits. J Infect Dis 2025; 231:e803–12. 10.1093/infdis/jiae631 PMC11998578 39693248

