# Supplementary material for: Sequential Time to Positivity as a Prognostic Indicator in Persistent Candidemia
Source: Open Forum Infect Dis. 2026 Oct 1;13(10):ofag619. doi: 10.1093/ofid/ofag619 (PMC13660687; doi:10.1093/ofid/ofag619)
Supplement: Supplementary file 1 [file ofag619_supplementary_data.docx]

**SUPPLEMENTARY MATERIAL**

**Figure S1.** Mortality rates stratified by the time-to-positivity ratio.

Abbreviation: TTP: time to positivity.

**TABLES**

**Table S1.** TTP in patients according to the source and management of candidemia.

| **Data** | **TTP [IQR]** |
| --- | --- |
| Patients with catheter-related candidemia (N = 31) |  |
| TTP of the first positive BC, (hours) | 23 [17 – 40.2] |
| TTP of the sequential positive FUBC, (hours) | 40 [32 – 57] |
| TTP ratio | 1.5 [1.3 – 2.4] |
| Patients with catheter-related candidemia and source control within 24h of BC collection (N = 31) | |
| TTP of the first positive BC, (hours) | 20.1 [14.6 – 40.7] |
| TTP of the sequential positive FUBC, (hours) | 32 [28 – 48] |
| TTP ratio | 1.5 [1.3 – 2.2] |
| Patients with other sources other than catheter-related candidemia (N = 38) | |
| TTP of the first positive BC, (hours) | 37 [21.6 – 47.1] |
| TTP of the sequential positive FUBC, (hours) | 47 [36 – 71] |
| TTP ratio | 1.2 [1.0 – 2.3] |

Abbreviations: IQR: interquartile range; TTP: time to positivity.

**Table S2.** Univariate logistic regression analysis of 30-day mortality according to the TTP ratio.

| **Parameter** | **Univariate OR 30-day mortality**  **(95% CI)** | ***P-value*** |
| --- | --- | --- |
| TTP ratio, per 0.1-unit increase | 0.98 (0.94 – 1.03) | 0.412 |

Abbreviations: OR: Odds ratio.

**Table S3.** Sensitivity analysis using different cutoff points to explore the association between TTP ratio and 30-day mortality.

| **TTP ratio cutoff** | **Comparison (reference)** | **Univariate OR 30-day mortality**  **(95% CI)** | **p-value** |
| --- | --- | --- | --- |
| P10 (0.8) | <P10 vs ≥P10 (ref.) | 1.2 (0.2–7.6) | 0.856 |
| P25 (1.1) | <P25 vs ≥P25 (ref.) | 1.6 (0.5–4.8) | 0.401 |
| P50 (1.4) | <P50 vs ≥P50 (ref.) | 1.6 (0.6–4.4) | 0.328 |
| P75 (2.3) | <P75 vs ≥P75 (ref.) | 1.5 (0.5–4.9) | 0.502 |
| P90 (3.7) | <P90 vs ≥P90 (ref.) | 3.1 (0.3–28.0) | 0.318 |

Abbreviations: OR: Odds ratio; TTP: time to positivity.
